# From Player to Pawn: Viral Avirulence Factors Involved in Plant Immunity

**DOI:** 10.3390/v13040688

**Published:** 2021-04-16

**Authors:** Changjun Huang

**Affiliations:** Key Laboratory of Tobacco Biotechnological Breeding, National Tobacco Genetic Engineering Research Center, Yunnan Academy of Tobacco Agricultural Sciences, Kunming 650021, China; cjhuang@zju.edu.cn; Tel.: +86-877-2075024

**Keywords:** plant viruses, plant immunity, NB-LRR, avirulence gene, effector-triggered immunity (ETI), viral effectors

## Abstract

In the plant immune system, according to the ‘gene-for-gene’ model, a resistance *(R)* gene product in the plant specifically surveils a corresponding effector protein functioning as an avirulence (*Avr*) gene product. This system differs from other plant–pathogen interaction systems, in which plant *R* genes recognize a single type of gene or gene family because almost all virus genes with distinct structures and functions can also interact with *R* genes as *Avr* determinants. Thus, research conducted on viral Avr-R systems can provide a novel understanding of *Avr* and *R* gene product interactions and identify mechanisms that enable rapid co-evolution of plants and phytopathogens. In this review, we intend to provide a brief overview of virus-encoded proteins and their roles in triggering plant resistance, and we also summarize current progress in understanding plant resistance against virus *Avr* genes. Moreover, we present applications of *Avr* gene-mediated phenotyping in *R* gene identification and screening of segregating populations during breeding processes.

## 1. Introduction

Plant viruses contain single-stranded or double-stranded RNA or DNA genomes and vary substantially in their genome structure and organization. Moreover, limited viral genome sizes and coding capacities have resulted in evolution of multifunctional proteins that are involved in different steps in the virus life cycle, including replication, movement, encapsidation and transmission. On the other hand, as obligate intracellular parasites, plant viruses absolutely depend on the host cell machinery to multiply, move throughout the plant and spread to susceptible hosts. During infection, viruses consume a substantial amount of host resources; subsequently, disease symptoms develop as a consequence of disruptions of the cellular machinery required for plant physiology and natural growth, and these disruptions eventually result in developmental abnormalities and other phenotypic manifestations. Viruses can be critical players in pathogenesis through direct or indirect interactions. However, in some plant species or varieties, virus-encoded proteins can sometimes act as determinants in plant defense responses and as host-controlled pawns to elicit extreme resistance (ER).

According to the zigzag model of plant–pathogen interactions, the plant innate immune system is broadly divided into two different layers: pathogen-associated molecular pattern (PAMP)-triggered immunity (PTI) and effector-triggered immunity (ETI) [1]. PTI is activated by specific recognition between PAMPs, such as bacterial flagellin and fungal chitin, and the corresponding membrane-anchored pattern recognition receptors (PRRs) of plants, which serve as the first layer of defense against invading pathogens. Plant viruses were historically viewed as non-PAMP coding pathogens and plant antiviral immunity was previously excluded from classical PTI models. However, recent evidence shows that PTI also operates against viruses in plants: For example, novel paradigms in antiviral immunity include (I) identification of dsRNAs and viral nucleic acids as PAMPs [2,3]; (II) plant virus effects on cell wall remodeling that imply that virus infections can modulate damage-associated molecular patterns (DAMPs) pathways with molecular mechanisms similar to PTI [4,5,6,7]; (III) several PRRs, e.g., NIK1, BAK1, BIR1, BKK1 (BAK1-Like 1) and Serk1 that have been shown to have roles during antiviral PTI [8,9,10,11,12]; and (IV) virus encoded proteins that interact with host factors involved in PTI pathways and interfere with PTI-mediated signaling to activate effector-triggered susceptibility (ETS) [13,14,15,16,17,18].

On the other hand, to counteract ETS, plants have evolved intracellular resistance (R) proteins that directly or indirectly recognize pathogen effectors or Avr factors to activate ETI and trigger the second layer of defense. ETI is often manifested as a hypersensitive response (HR), characterized by rapid cell death, production of reactive oxygen species (ROS) and salicylic acid (SA) induction and expression of defense-related genes [19,20,21]. Since the first viral Avr factor and antiviral *R* genes were identified in 1984 [22] and 1994 [23], increasingly diverse Avr factors and R proteins have been characterized in different virus–plant combinations. Most R proteins contain a nucleotide binding and leucine-rich repeat domain (NB-LRR) with an N terminal coiled coil domain (CC) or Toll/interleukin-1 receptors (TIR) domains. Increasing evidence also substantiates the notion that plants deploy typical ETI-based innate immune systems to control virus infections. The aim of this review is to summarize numerous advances about viral Avr factors and their roles in plant immunity.

## 2. Coat Proteins (CP)

The CP, also known as the capsid protein, encapsidates and protects viral genomes from damage. Early expressed CPs function in disassembly of parental virions and have roles in assembly of progeny virions during the final infection steps. However, more and more evidence has shown that CPs of all plant viruses are multifunctional and have various roles during different replication stages, ranging from early to late events in the infection cycle. The diversity of these functions in different viral systems includes virus transmission by specific vectors, translation of viral RNA, regulation of intercellular and systemic movement of the virus, suppression of both post transcription gene silencing (PTGS) and transcription gene silencing (TGS), as well as determination of symptomatology and pathogenesis [24,25]. Owing to their obvious importance, CPs were the first example of pathogen-derived transgene resistance in plants [26]. In fact, CP encoded transgenic resistance provides an excellent solution to the global viral problems and provides an important venue for both basic and applied disease resistance breeding research and crop production [27].

Compared to CP-mediated genetic resistance engineered within the last 30 plus years, CP-induced natural resistance has evolved over millions of years. The role of CPs in the activation of *R* gene-mediated host defenses has been extensively characterized. The CPs of *Tobacco mosaic virus* (TMV), *Tomato mosaic virus* (ToMV), *Tobacco mild green mosaic virus* (TMGMV), *Bell pepper mottle virus* (BPeMV), *Paprika mild mottle virus* (PaMMV), *Obuda pepper virus* (ObPV), *Pepper mild mottle virus* (PMMoV), *Potato virus X* (PVX) and *Mungbean yellow mosaic virus* (MYMV) each serve as Avr factors that elicit resistance controlled by cognate dominant *R* genes (Table 1).

TMV CP was identified as an Avr responsible for eliciting host ER responses during interactions with the *Nicotiana sylvestris N′* gene. Two groups independently found this property by analyzing a series of recombinant viruses between resistance-inducing (RI) and resistance-breaking (RB) strains [28,29,30]. Subsequently systematic studies of CP amino acid substitutions have demonstrated that *N′*-mediated recognition requires maintenance of the CP three-dimensional structure, either directly, or through specific structural motifs [31,32]. The *N′* gene and its orthologues were recently cloned from *N. sylvestris* and other TMV resistant *Nicotiana* species and shown to encode CC-NB-LRR type proteins [33,34]. Interestingly, a more recent study of phylogeny of the CP of tombusviruses indicated that CP representatives of the family could be divided into four clades. All tested CP members in two separate clades triggered an HR in *Nicotiana* section *Alatae* species [35]. Moreover, a previous study had shown that several members of *Nicotiana* section *Alatae* carry functional *N′* orthologues [34] and implied that *N′* and *N′* orthologues might have been inherited from a common ancestor followed by evolution to confer tobamovirus and tombusvirus resistance to *Nicotiana* genus species. In pepper, a broadening spectrum of resistance to seven known pepper-infecting species of tobamoviruses (TMV, ToMV, TMGMV, BPeMV, PaMMV, ObPV and PMMoV) is conferred by the corresponding *localization (L)* alleles [36]. *L* gene alleles also encode CC-NB-LRR type resistance proteins with the ability to elicit resistance responses to different tobamoviral CP Avr effectors [37,38]. Since both *N′* and its *Nicotiana* orthologues and the *L* alleles from pepper mediate resistance against tobamoviruses by recognizing the CP [33,34], it seems that these genes have evolved from a common *Solanum* ancestor. However, a resistance gene evolution assay indicates that the *L* gene from pepper is not an *N′* orthologue, suggesting that tobamovirus resistances in pepper and *Nicotiana* originated independently [34]. Several *R* genes within the same locus recognize different CP proteins from overlapping virus species indicating that the conserved R proteins are able to recognize similar structures but with an adapted spectrum. These results also support the idea that interactions between *L* genes or *N′* orthologues and tobamovirus CPs serve as good systems for study the mechanisms and evolution of virus perception by plants.

Another representative study involves PVX CP-elicited ER mediated by the *Rx1* gene, which encodes a class of CC-NB-LRR R proteins in potato [39]. Under virus-free conditions, intramolecular interactions between the CC domain and NB or LRR domains retain *Rx1* in an auto-inhibited (off) state [79,80]. Upon PVX infection, Rx1 protein recognizes the PVX CP by leucine-rich repeat domain interactions that result in disruption of Rx1 intramolecular host interactions. However, PVX CP-induced ER by Rx1 does not involve natural cell death at the inoculation site, but instead suppresses virus replication per se, even in protoplast infections. In contrast, *RX1* does trigger an HR upon overexpression of the Avr PVX CP or under high PVX concentrations [40]. An additional study has demonstrated that nuclear-cytosolic shuttling of CP-activated Rx1 mediated by Ran GTPase-activating protein 2 (RanGAP2) is required for PVX defenses [81]. However, recent studies have shown that nuclear or cytosol restricted Rx1 variants cannot trigger ER or suppress the spread of virus infections, but can still induce an HR. Furthermore, perturbation of the nucleocytoplasmic distribution of *Rx1* leads to translational arrest of PVX CP transcripts and compromises extreme resistance against PVX [82]. Thus, many mysteries still need to be addressed, e.g., how to explain the mutual regulation of the PVX CP and NLR activation and whether other important host factors are involved in ER and HR induction. Notably, another ER gene locus in potato, *Nx*, also confers resistance to the PVX CP. However, ER by the *Rx1* gene is induced via interactions with PVX CP conserved amino acids (aa) 121–127, whereas the *Nx* gene confers resistance to PVX through the recognition of PVX CP aa 62–78 [39,42,83].

In the *Arabidopsis thaliana* ecotype Di-17, *Turnip crinkle virus* (TCV) CP P38 functions as the Avr resistance determinant for the HRT CC-NB-LRR R protein [46,84]. By use of natural mutant isolates and interaction region screening, the TCV P38 *N* terminus has been shown to be involved in eliciting resistance responses [84,85]. Moreover, the TCV P38 *N* terminal nuclear localization domain is important for elicitation of host resistance responses and could be a key trigger for HRT-dependent resistance to TCV [86]. In another *A. thaliana* ecotype C24, *RCY1*, which encodes a CC-NB-LRR class R protein, was isolated and identified as the first *R* gene conferring resistance to *Cucumber mosaic virus* (CMV) [44]. The CMV genes involved in the *RCY1* Avr resistance response also mapped to the CP [45]. Interestingly, both the *RCY1* and *HRT R* genes belong to HRT/RPP8 gene family, and the *RCY1* locus in ecotype C24 were was found to be allelic to *HRT* in Dijon-17. These functionally divergence genes therefore seem to have evolved via recombination of ancestral genes [87]. More intriguingly, the CMV and TCV CPs, which lack sequence similarity, recognize the allelic *RCY1* and *HRT* genes [45,84]. Therefore, several possibilities have been presented; one possibility is that *RCY1* and *HRT* are elicited by completely different ligands. Another possibility is that the single CPs of CMV and TCV, or their possible complexes with other host components (e.g., guardee or decoy factor) may have highly similar CP protein folding domains [44].

## 3. Replication-Related Proteins

Replication of viruses in cells by use of their genetic information to commandeer host cell components and machinery is a major feature that distinguishes viruses from other pathogens. Plant DNA viruses, such as geminivirus, replicate by association with cellular DNA-dependent DNA polymerases in infected cell nuclei and formed minichromosomes [88]. In contrast, RNA replication results in rearrangements of intracellular membranes and frequently induces the formation of vesicles that contain RNA-dependent RNA polymerases and genomic RNA [89]. To replicate their genomes, viruses usurp host factors that interact with viral gene products via protein–protein or protein–nucleic acid interactions. One of the best studies using a yeast in vivo replication system has identified more than 250 host factors that interact with viral replicase, replication proteins, or the viral RNA to affect replication and recombination of TBSV [90]. In addition, large sets of data have demonstrated that both DNA and RNA virus replication-related proteins can elicit *R* gene-driven effector triggered immunity (ETI) causing HR (Table 1).

*Ty-2* is a major source of dominant resistance against *Tomato yellow leaf curl virus* (TYLCV) that has been widely employed in tomato breeding programs that have released numerous commercial cultivars. Recently, *Ty-2* was cloned and shown to encode a CC-NB-LRR protein [48]. Using agroinfiltration to transiently co-express *Ty-2* along with the *Rep/C1* and *C4* genes of the RI and RB TYLCV strains, respectively, in *N. benthamiana*, only the RI TYLCV strain Rep/C1 protein consistently elicited a HR when co-expressed with the *Ty-2* protein. This result clearly indicates that the Rep/C1 protein is the *Avr* determinant triggering the *Ty-2* based (strain-specific) resistance response [48].

As the first and one of the best studied plant virus resistance genes, the *N* gene was cloned almost twenty years ago and shown to be a member of the TIR-NBS-LRR class of plant disease resistance genes [23]. Subsequent efforts focused on identification of the viral Avr factor for the *N* gene. Beachy’s group initially reported a role for the 126/183-kDa replicase, but not the CP or MP, in induction of HR in tobacco containing the *N* gene [91], Later, they showed that the 126-kDa protein sequence containing the methyltransferase and helicase-like domains, but not the 183-kDa readthrough protein containing the polymerase domain, is the Avr factor that triggers *N*-mediated defense responses [92]. Ultimately, Barbara Baker’s team demonstrated that expression of the 50-kDa TMV helicase fragment (p50) of the 126-kDa replicase protein is sufficient to elicit *N*-mediated ER [49]. They also showed that HR induction depends on features of the p50 protein that are independent of its ATPase/helicase activity. Similar HR characteristics induced by avirulent replicases that are not dependent on the enzymatic activity of the protein also have been observed in the 2a polymerase of CMV in cowpea plants [53]. The amino acid mutants that alter the highly conserved polymerase Gly-Asp-Asp (GDD) motif abolish replicase activity; however, these mutants do not affect HR induction in cowpea plants. 

The *Pvr4* gene encodes a broad-spectrum CC-NBS-LRR type resistance protein known to elicit resistance against multiple potyviruses, including *Pepper mottle virus* (PepMoV), *Pepper severe mosaic virus* (PepSMV) and *Potato virus Y* (PVY) in *Capsicum annuum* [50]. Kim et al. used agrobacterium transient expression of potyvirus coding regions and showed that the PepMoV, PepSMV and PVY RdRp NIbs proteins serve as Avr factors that elicit *Pvr4* resistance in pepper plants [51]. The cylindrical inclusion (CI) protein of the potyvirus contains RNA helicase activities and are essential for genomic RNA replication. In the brassica–TuMV interaction system, the TuMV CI protein has been identified as the viral *Avr* determinant for two resistance genes, *TuRB01* and *TuRB05*, in the AA subgenome of *Brassica napus* [55,56].

## 4. Movement Proteins (MP)

Virus cell-to-cell and long distance movement throughout the plant from initial infection sites are controlled by specific viral MPs. Generally, plant virus movement is divided into three steps: (I) intracellular movement in which virus is trafficked along different organelles within a single cell from the sites of replication to the plasmodesmata [93]; (II) intercellular movement involving transport of virus through plasmodesmata (PD) cell-wall structures serving as cytoplasmic connections between plant cells [94]; (III) systemic movement throughout the plant when viruses transit through the vascular system to distal leaves, roots and occasionally to reproductive organs [95,96]. Although different MPs have been shown to use various pathways and mechanisms for virus transport, numerous host factors and viral proteins may be required for each movement step.

Several examples have shown that viral MPs can elicit an HR response via interactions with *R* gene products. One of the best-characterized systems is the interaction between the *Tomato spotted wilt virus* (TSWV) NSm movement protein with the tomato *Sw-5b* and tobacco *RTSW* resistance gene proteins. Sw-5b belongs to the CC-NB-LRR type immune receptors and contains an extended N-terminal Solanaceae domain (SD) [57,58]. This R protein confers broad-spectrum resistance to various American-type orthotospoviruses but not to the Euro-Asian-type orthotospoviruses [57,97,98]. Two groups independently demonstrated at almost same time that Sw-5b resistance is triggered by the NSm cell-to-cell movement protein [59,60]. Subsequent work by Tao’s group showed that a 21-aa peptide region positioned at aa 115–135 in TSWV NSm (NSm^21^), which is highly conserved among the American-type orthotospoviruses, but not the Euro-Asian-types, is sufficient to trigger Sw-5b-mediated HR [97,99]. In addition, the group also found that Sw-5b NLR adopts a two-step recognition mechanism to enhance NSm perception. In addition to direct interactions between the LRR domain and NSm or NSm^21^, the SD domain functions as an extra sensor to detect low levels of NSm or NSm^21^ and enhance resistance of Sw-5b [100,101]. *RTSW*, another *R* locus from wild tobacco *N. alata* that confers ER to TSWV, has been introgressed into cultivated tobacco. By using two different transient expression systems, we showed that the NSm protein of TSWV acts as an *Avr* determinant of *RTSW*-based resistance [61]. Moreover, both our results and those of Tao’s group showed that intercellular trafficking of NSm can be uncoupled from its HR function in the induction of RTSW and Sw-5b resistance [61,102]. More importantly, our evidence indicates that although *RTSW* and *Sw-5b* behave as single dominant genes that confer ER by interacting with the same NSm protein encoded by the same virus, they recognize different elicitor domains (EADs) [61]. The same pattern also has been shown to confer resistance to ToMV in tomato harboring the *Tm-2* and *Tm-2^(2)^* genes. The 30-KDa MP of ToMV elicits both *Tm-2* and *Tm-2^(2)^* resistance, but has different interacting domains [62] because the EADs map to the MP N- and C-termini, respectively [103]. 

The model grass *Brachypodium distachyon* Bd3-1 inbred line harbors a resistance gene designated *Bsr1* that interacts with the *Barley stripe mosaic virus* (BSMV) ND18 triple gene block 1 (TGB1) movement protein to elicit ER during infection. Lee et al. have shown that two amino acids within the protein are required to elicit resistance, and that the BSMV Norwich strain has a two amino acid changes in TGB1 that abrogates necrosis to enable systemic infections [63]. In potatoes, the 25-kDa MP of PVX elicits *Nb*-Mediated ER, and that an isoleucine residue at position 6 of the MP protein is required for activation of the *Nb* response [68]. More intriguingly, recent evidence has revealed that the PVX MP functions as a silencing suppressor by directly targeting AGO1 degradation through the proteasome pathway [104]. Therefore, more systematic studies need to be conducted on this multifunctional protein to clarify the roles of important sites or domains in nucleotide binding, RNA helicase activity, plasmodesmatal gating, suppressor activity, etc.

## 5. RNA Silencing Suppressors (RSS)

RNA silencing (also known as RNA interference, RNAi) functions in endogenous gene regulatory and exogenous antiviral mechanisms in plants [105]. As a counterdefense, viruses have evolved RSS proteins that function to inhibit RNA silencing through diverse mechanisms [106,107]. The first mode of RSSs action is binding of long dsRNA or siRNA duplexes that arise during viral infections to inhibit siRNA biogenesis or RISC formation [108]. A second mode of action is binding to important components in the silencing pathway [109,110]. Recently, RNA silencing has been regarded as pattern-triggered immunity (PTI) against viruses and dsRNAs during the replication of RNA and DNA viruses and has been categorized as a pathogen-associated molecular patterns (PAMP) defense [111]. Consequently, by analogy of these steps in virus-host interactions with the steps outlined in the standard zigzag model, RSSs has been defined as a class of ETS that circumvents host RNA silencing and counters against the first layer of plant antiviral defenses [3,112,113]. Given the importance of suppressing RNA silencing for virus survival, it is not unexpected that direct and indirect interactions would occur between RSSs and *R* genes to elicit ETI, which acts as a second plant defense layer [107]. 

The orthotospoviral NSs silencing suppressor protein is a well characterized RSS that conducts local and systemic silencing suppression functions by binding small and long dsRNAs [114,115]. Recently, NSs has been identified as the *Avr* determinant for *Tsw*-based resistance in pepper [69]. Considering the role of NSs to counter defenses against RNA silencing, further dissections were tested by screening a large set of mutants for lost RI activity or silencing suppression. This result supports the idea that the NSs protein has evolved to fine tune and uncoupled Avr and RSS functions [116]. Recent studies tracing HR induction lineages and silencing suppression associated with the CPs of the *Tombusviridae* also support the conclusion that SSR and HR elicitation involve independent motifs. Among the four clades in the phylogenetic tree of 54 CPs tombusvirids, two separate CP clades triggered an HR in *Nicotiana* species of section *Alatae* but did not have silencing suppressor activity, whereas in one clade the CP functioned as an SSR, but lost the capacity to trigger HR in *Nicotiana* species [35].

The Polerovirus P0 protein is another well-known SSR that can mediate degradation of ARGONAUTE1 (AGO1), one of the most important components in RNA silencing and antiviral responses [117]. The *N. glutinosa RPO1* gene elicits ER against poleroviruses *Turnip yellows virus* (TuYV) and *Potato leafroll virus* (PLRV), and is inherited as a single, dominant resistance allele. The P0 proteins from TuYV, PLRV and *Cucurbit aphid-borne yellows virus* (CABYV) were found to elicit an HR in *N. glutinosa* accessions containing the *RPO1* locus. To dissect important roles of the P0 F-box motif in autophagic degradation of AGO1 proteins, motif mutants were constructed to test requirements for elicitation of RPO1-mediated HR. The results showed that P0 mutants with substitutions in the F-box motif that abolished SSR activity were unable to elicit HR, suggesting that HR induction requires a functional P0 protein [70]. In cotton (*Gossypium hirsutum*), *Cbd* is a single dominant gene conferring ER to the polerovirus, *cotton leafroll dwarf virus* (CLDV). Agrofoglio and coworkers characterized the SSR activity of the P0 proteins encoded by wildtype (WT) CLRDV and a RB CLRDV isolate and evaluated the roles of these proteins in breaking *Cbd*-resistance in cotton plants. They found that WT and RB CLRDV P0s behave similarly during RNA silencing interference; however, RB CLRDV P0 enabled WT CLRDV and a chimeric infectious clone to systemically infect *Cbd*-resistant cotton varieties. Therefore, the CLRDV P0 protein is also an *Avr* determinant that functions in *Cbd*-based resistance [71].

## 6. Other Proteins

In contrast to a simple classification in which ER caused by viral elicitors falls into the four categories above, research within the past few years shows that ER determinants are largely spread throughout the viral genome [118,119]. Additional elicitors of viral proteins, with the exception of those discussed in the previous sections, have been shown to contribute to viral ETI resistance. For example, the first plant virus *avr* gene, P6 of CaMV was identified through its capacity to elicit an HR in *Datura stramonium* and *N. edwardsonii*, by dissecting a series of gene swaps between infectious clones of different strains of CaMV [22,72]. Subsequent discreet studies further showed that P6 elicits a non-necrotic resistance response in *N. glutinosa*, but segregated independently from the cell death responses of *N. edwardsonii* or *N. clevelandii* [120,121], although specific *R* genes affecting these processes have not been isolated. P6 has also been recognized as a versatile viral protein that has key roles in several steps of virus infections [122]. For example, P6 is a transactivator of translation, a central component of amorphous inclusion bodies, an RSS and a main determinant of host range and pathogenicity [72,122,123,124]. P6 has also been associated with virus movement, modulates host defenses including both PTI and ETI, and suppresses SA-dependent autophagy [15,16,125,126,127,128]. Although the overall distinction between Avr triggering and other biology functions of P6 remain obscure, subtle assays have showed that the role of P6 in ER elicitation can be uncoupled from its roles as a translational transactivator [129,130].

Ry_sto_ resistance, which confers ER against PVY and related viruses from the wild relative *S. stoloniferum*, has been widely employed in potato breeding programs as an importance resistance trait. Recently, the Ry_sto_ gene was cloned and found to encode a TIR-NB-LRR protein [76]. Nevertheless, the *Avr* determinant for Ry_sto_ was determined more than twenty years ago, when Mestre et al. demonstrated that the NIa proteinase (NIaPro) from PVY is an elicitor of Ry_sto_-mediated resistance [77]. In this case, transient expression of PVY NIaPro by agro-infiltration elicited a HR in Ry_sto_ resistant but not in ry_sto_ susceptible potato [77]. In a subsequent study, protease activity of NIaPro was shown to be required but not sufficient for elicitation of *Ry_sto_*-mediated potyviral resistance [78]. However, conflicting results appeared after the Ry_sto_ gene was isolated because similar transient expression strategies with different viral proteins from PVY and *Potato virus A* (PVA) indicated that Ry_sto_ recognizes the CP but not other proteins as Avr factors in Ry_sto_ transgenic *N. tabacum* plants [76]. Therefore, it remains to be determined whether the HR phenotype is elicited by transient expression of different viral proteins (NIaPro and CP) or results from different interactions in the two experimental hosts (potato and tobacco).

In soybean plants (*Glycine max*), the *Rsv1* gene confers resistance to the N strain of *soybean mosaic virus* (SMV), one of the most devastating potyviruses that causes huge economic losses in soybean production worldwide. Through construction of a series of chimeras and site-directed mutants, Eggenberger and colleagues mapped SMV P3 and helper-component proteinase (HC-Pro) components involved in elicitation of *Rsv1*-mediated ER. Since P3 and HC-Pro are cistrons within a read-through protein, it seems that HC-Pro and P3 are recognized by *Rsv1*-mediated defense responses as a precursor polyprotein [73]. More recent research revealed that the complexity of the *Rsv1* locus may be a reason why the recognition sites are distributed throughout the HC-Pro and P3 cistrons. Multiple distinct resistance genes, likely belonging to the NB-LRR class, have been discovered within the *Rsv1* locus [131]. In addition, the P3 protein alone was found to be an *Avr* determinant for two dominant resistance genes, *TuRB03* and *TuRB04*, in the brassica-TuMV interaction system [56,74].

## 7. Application of Viral Avr Factors in Resistance Studies

### 7.1. Agrobacterium Co-Infiltration Transient Expression as a Tool for Isolation and Identification of Disease Resistance Genes

In early studies, viral Avr factor identification was conducted via several approaches including gene swaps between resistance and susceptible virus strains or mutants, and ectopic expression of Avr candidate genes within a virus vector. Subsequently, agroinfiltration has been shown to be a more convenient and powerful tool for screening of viral proteins capable of triggering HR in R-containing plant hosts. According to the ‘gene-for-gene’ theory, one *R* gene product in the plant specifically perceives a matching effector protein, and a definitive *Avr* gene also can be inversely employed to screen for corresponding *R* genes. Several successful case studies have demonstrated the utility and operability of this approach [41,132]. In contrast to Avr factor screening, in which single *Avr* candidate genes are agroinfiltrated into resistance host plants, *R* gene screening is conducted by mixing agrobacterium strains carrying the candidate *R* gene and the *Avr* genes, and the mixtures are co-infiltrated into susceptible host plants such as *N. benthamiana*. Compared to the several months required for transgenic assays or gene knockout assays, agrobacterium co-infiltration transient expression systems just require two or three days. Previous experiments have confirmed that *Rx2* (AC15 clone) and *RP28* genes can be easily and quickly isolated from a 200 Rx homologue library from potato and a 99 candidate *R* gene library from pepper by using Agrobacterium coinfiltration approaches [41,132]. Therefore, the Agrobacterium approach is a handy tool that is less time consuming and labor intensive than the more traditional methods. The Agrobacterium system could also provide a convenient method to identify disease resistance genes because *R* genes encoding NB-LRR type receptors are frequently members of large gene families, organized in complex clusters of paralogous genes in the plant genome. Of course, several additional candidate R homologues still may need to be cloned and identified even if located within a very narrow chromosome fine mapping region.

### 7.2. Avr Gene-Based Diagnostic Approaches for Allele Identification and Phenotyping

Identification of new candidates for virus resistance is a prerequisite for effective utilization of diverse germplasm in breeding programs. Multiple alleles arising by rapid evolution through tandem and segmental gene duplications, recombination, unequal crossing-over or point mutations are common within *R* gene families. The high sequence similarity of encoding and flanking regions of *R* gene alleles by marker assisted genotyping are huge challenges. However, coevolution and diversified selection of *R* genes and the pathogen’s Avr effectors often result in multiple recognition regions between a single *R* gene with each allele and corresponding species or strain-specific Avrs. Several examples of allelic series of virus *R* genes are known in plants; for example the *L*^1^, *L*^2^, *L*^3^, and *L*^4^ alleles confer broad spectrum of resistance to different tobamoviruses by recognizing their CP elicitors [36]. Therefore, HR phenotyping by using specific recognition between R and Avr proteins represents an alternative approach to screen different alleles from germplasm variants. In this regard, *Avr* gene-based phenotyping has been established and applied to rice blast disease resistance identification in the field by using different Avr factors [133].

For fine mapping and cloning of virus resistance genes, a precise disease resistance test is a prime requirement for resistance identification in segregation populations, e.g., F2, BC, RIL or NIL populations. Traditional virus disease resistance tests performed in mapping or breeding programs involves virus infectious clones, insect vectors, or sap mediated virus inoculations. However, the obvious shortcomings of these methods, including low accuracy, complicated operations, time consuming experiments, and labor intensive trials has limited their wide application. However, our experience has indicated that *Avr* gene-based diagnostic approaches can provide rapid disease resistance tests that overcome limitations of virus sap inoculations and greatly improve the efficiency and accuracy of test plant inoculations [61].

## 8. Conclusions and Perspectives 

Over the past three decades, our knowledge about plant virus resistance genes and corresponding Avr factors has advanced dramatically. Compared to other pathogens (i.e., bacterial, fungal and oomycetes), viral *Avr* gene identification is relatively easy due to small virus genome sizes and limited numbers of gene products. Most virus *Avr* genes have been matched with an *R*-gene type NB-LRR receptor (Table 1). However, *Avr* genes that elicit activity of some mapped or cloned antiviral *R* genes have not yet been identified. These include the *I* gene in *Phaseolus vulgaris* that confers resistance to multiple potyviruses [134], *Y-1* in *S. tuberosum* resistance to PVY [135], *Pv1* and *Pv2* in *Cucumis melo* resistance to *Papaya ringspot virus* [136], *Ctv* resistance in *Poncirus trifoliate* to *Citrus tristeza virus* [137] and *BcTuR03* resistance in *B. campestris* to TuMV [138]. Thus, further studies need to be conducted to identify Avr factors and their roles in eliciting resistance and functions in virus multiplication.

Previous studies with virus Avr-R interactions have provided new insights and paved the way to plant innate immunity studies of other pathogens. Among these are investigations of direct interactions between NSm (or NSm^21^) and Sw-5b that revealed a novel two-step recognition mechanism involving the SD and LRR domains of Sw-5b [97,100], indirect interactions between the *N* gene and TMV p50 mediated by an intermediate protein NRIP1 [139] and recognition of the TMV MP by Tm-2^(2)^ involving an intermediate NbMIP1 [140,141]. These results indicate that associations between NLRs and their corresponding effectors may not be sufficient to directly activate R protein defense responses, but may require additional molecular partners. In contrast to the well characterizations of TSWV Nsm/Sw-5b, TMV p50/N, PVX CP/Rx1, ToMV MP/Tm-2^(2)^, other Avr and R proteins interactions were limitedly characterized experimentally. Thus, a major challenge for the future is to identify the precise interaction mechanisms between viral Avr factors and their matching NB-LRR type receptors, as it is possible such multi host interactions are common. The resolution of such mechanisms may require more powerful technological tools to detect and analyze subtle signaling complexes. Nevertheless, several novel experimental approaches, including TurboID-based proximity labeling, high throughput omics analyses, protein–protein interaction networks and machine learning technologies have recently demonstrated their potential for comprehensive understanding of complex biological process such as Avr/R protein signaling cascades [142,143].

Notably, recent cryo-EM structural and functional analyses of CC-NB-LRR (ZAR1) and TIR-NB-LRR (ROQ1 and RPP1) proteins have shed new light on resistosome signaling mechanisms and provide an excellent template for other CC- and TIR-NB-LRR protein functional cascades [144,145,146,147]. It would be interesting to discover whether other CC-NB-LRR proteins self-associate and oligomerize into larger structures to form membrane pores since previous results have revealed distinct subcellular localization of R proteins. For instance, nucleocytoplasmic shuttling requirements of the PVX CP activated Rx1[81], Tm-2^(2)^ functions on the plasma membrane [140] and Sw-5b exhibits nucleocytoplasmic localization patterns in the absence of virus infections or NSm induction [139,148]. It also would be worthwhile to determine whether other TIR-NB-LRR proteins form tetrameric assemblies that act as holoenzymes to mediate NAD^+^ hydrolysis that can trigger plant immunity responses. Future steps will require advances in structural studies, understanding patterns and mechanisms of R protein recognition and activation of viral Avr factors. Addressing such fundamental mechanisms will clarify major gaps in our understanding of plant virus defense networks.

## Figures and Tables

**Table 1:** Plant virus avirulence (Avr) factor and cognate NB-LRR resistance genes.

*Avr* Gene	Virus Species	*R* Gene (Type)	Host Plant	Reference
Coat Protein (CP)				
CP	*Potato virus X* (PVX)	*Rx1* (CC-NBS-LRR)	*Solanum tuberosum*	[39,40]
CP	PVX	*Rx2* (CC-NBS-LRR)	*S. tuberosum*	[41]
CP	PVX	*Nx* (locus)	*S. tuberosum*	[42]
CP	*Tobacco mosaic virus* (TMV)	*N′* (CC-NBS-LRR)	*Nicotiana sylvestris*	[29,30,33]
CP	TMV, *Tomato mosaic virus* (ToMV), *Tobacco mild green mosaic virus* (TMGMV), *Bell pepper mottle virus* (BPeMV), P*aprika mild mottle virus* (PaMMV), *Obuda pepper virus* (ObPV), *Pepper mild mottle virus* (PMMoV), *Mungbean yellow mosaic virus* (MYMV)	*L^1-4^* (CC-NBS-LRR)	*Capsicum annuum*	[36]
CP	MYMV	*CYR1* (CC-NBS-LRR)	*Vigna mungo*	[43]
CP	*Cucumber mosaic virus* (CMV)	*RCY1* (CC-NB-LRR)	*Arabidopsis thaliana*	[44,45]
P38	*Turnip crinkle virus* (TCV)	*HRT* (CC-NB-LRR)	*A. thaliana*	[46,47]
Replication-Related Protein				
Rep/C1	*Tomato yellow leaf curl virus* (TYLCV)	*Ty2* (CC-NB-LRR)	*S. habrochaites*	[48]
p50	TMV	*N* (TIR- NB-LRR)	*N. glutinosa*	[23,49]
RNA-dependent RNA polymerase (NIb)	*Pepper mottle virus* (PepMoV), *Pepper severe mosaic virus* (PepSMV), and *Potato virus Y* (PVY)	*Pvr4* (CC-NBS-LRR)	*C. annuum*	[50,51]
2a	CMV	*RT4-4* (TIR-NB-LRR)	*Phaseolus vulgaris*	[52]
	CMV	Unknown	*Vigna unguiculata*	[53,54]
Helicase (CI)	*Turnip mosaic virus* (TuMV)	*TurB01* (locus) *TurB05* (locus)	*Brassica napus*	[55,56]
Movement Protein (MP)				
NSm	T*omato spotted wilt virus* (TSWV), *Tomato chlorotic spot virus* (TCSV), *Groundnut ringspot virus* (GRSV), *Chrysanthemum stem necrosis virus* (CSNV) and *Impatiens necrotic spot virus* (INSV)	*Sw-5b* (SD-CC-NB-LRR)	*S. peruvianum*	[57,58,59,60]
NSm	TSWV	*RTSW* (locus)	*N. alata*	[61]
30-KDa MP	TMV, ToMV	*Tm-2* and *Tm-2^(2)^* (CC-NB-LRR)	*S.peruvianum*	[62]
TGB1	*Barley stripe mosaic virus* (BSMV)	*Bsr1* (CC-NB-LRR)	*Brachypodium distachyon*	[63]
BV1	*Bean dwarf mosaic virus* (BDMV)	*PvVTT1* (TIR-NB-LRR)	*P. vulgaris*	[64,65,66]
P1	*Cauliflower mosaic virus* (CaMV)	CAR1 (locus)	*A.thaliana*	[67]
25-KDa MP	PVX	*Nb* (locus)	*S. tuberosum*	[68]
RNA Silencing Suppressor (RSS)				
NSs	TSWV	Tsw (CC-NBS-LRR)	*C. annuum*	[50,69]
P0	*Cucurbit aphid-borne yellows virus* (CABYV), T*urnip yellows virus* (TuYV) and *Potato leafroll virus* (PLRV)	*RPO1*(locus)	*N. glutinosa*	[70]
P0	*Cotton leafroll dwarf virus* (CLRDV)	*Cbd* (locus)	*Gossypium hirsutum*	[71]
Other Proteins				
P6	CaMV	Unknown	*Datura stramonium and N. edwardsonii*	[22,72]
P3 + HC-Pro	*Soybean mosaic virus* (SMV)	*Rsv1* (CC-NBS-LRR)	*Glycine max*	[73]
P3	TuMV	*TurB03* (locus) *TurB04* (locus)	*B. napus*	[67,74,75]
NIaPro or CP?	PVY *Potato Virus A* (PVA)	*Ry_sto_* (TIR-NB-LRR)	*S. stoloniferum*	[76,77,78]
